# Dynamic Changes in Yeast Phosphatase Families Allow for Specialization in Phosphate and Thiamine Starvation

**DOI:** 10.1534/g3.118.200303

**Published:** 2018-05-10

**Authors:** John V. Nahas, Christine L. Iosue, Noor F. Shaik, Kathleen Selhorst, Bin Z. He, Dennis D. Wykoff

**Affiliations:** * Department of Biology, Villanova University, Villanova, Pennsylvania; † Department of Biology, University of Iowa, Iowa City, Iowa

**Keywords:** *Candida glabrata*, thiamine metabolism, phosphate metabolism, gene duplication and loss, acid phosphatase, parallel evolution

## Abstract

Convergent evolution is often due to selective pressures generating a similar phenotype. We observe relatively recent duplications in a spectrum of *Saccharomycetaceae* yeast species resulting in multiple phosphatases that are regulated by different nutrient conditions – thiamine and phosphate starvation. This specialization is both transcriptional and at the level of phosphatase substrate specificity. In *Candida glabrata*, loss of the ancestral phosphatase family was compensated by the co-option of a different histidine phosphatase family with three paralogs. Using RNA-seq and functional assays, we identify one of these paralogs, *CgPMU3*, as a thiamine phosphatase. We further determine that the 81% identical paralog *CgPMU2* does not encode thiamine phosphatase activity; however, both are capable of cleaving the phosphatase substrate, 1-napthyl-phosphate. We functionally demonstrate that members of this family evolved novel enzymatic functions for phosphate and thiamine starvation, and are regulated transcriptionally by either nutrient condition, and observe similar trends in other yeast species. This independent, parallel evolution involving two different families of histidine phosphatases suggests that there were likely similar selective pressures on multiple yeast species to recycle thiamine and phosphate. In this work, we focused on duplication and specialization, but there is also repeated loss of phosphatases, indicating that the expansion and contraction of the phosphatase family is dynamic in many Ascomycetes. The dynamic evolution of the phosphatase gene families is perhaps just one example of how gene duplication, co-option, and transcriptional and functional specialization together allow species to adapt to their environment with existing genetic resources.

Gene duplication is a major driver of diversity (Taylor *et al.* 2001; Zhang 2003; Drea *et al.* 2006; Scannell *et al.* 2007; Voordeckers *et al.* 2015; Dujon and Louis 2017). By generating raw material for natural selection, gene duplication may allow neo- or sub-functionalization, which could facilitate species’ adaptation to their particular environment. Specialization of duplicates can allow for improvement of individual functions that might have constrained each other in the ancestral gene (subfunctionalization) (Baker *et al.* 2013), or can allow for one of the paralogs to acquire a new function (neofunctionalization) (He and Zhang 2005; Conant and Wolfe 2008). Duplicates that do not provide a selective advantage are generally lost and there is evidence for this in numerous studies (Lynch and Conery 2000; Scannell *et al.* 2006; Naseeb *et al.* 2017). In *Saccharomyces cerevisiae*, there are a number of gene families that have arisen from multiple duplication events that allow for specialization, including flocculation and adhesion genes (de Groot *et al.* 2008; Van Mulders *et al.* 2010; de Groot *et al.* 2013), phosphatase genes (Tait-Kamradt *et al.* 1986; Venter and Hörz 1989; Orkwis *et al.* 2010), and cyclins (Wolfe and Shields 1997; Carroll and O’Shea 2002). In some cases, duplications and/or specializations exhibit convergence -*i.e.* the same characteristics in divergent species that were likely not in the ancestor, and thus, arose independently (Stern 2013). For example, *S. cerevisiae* and *S. pombe* each have three mating type gene cassettes that are understood to have arisen from independent gene duplication events, suggesting that this genetic architecture was adaptive (Klar *et al.* 2014; Hanson and Wolfe 2017; Dujon and Louis 2017).

During our studies with *Candida glabrata*, we identified a three-gene family of phosphatases (*CgPMU1-3*) that encodes phosphatases of differing enzyme specificity (Orkwis *et al.* 2010; Orlando *et al.* 2015). We previously identified *CgPMU2* as the phosphate-starvation inducible acid phosphatase gene that is analogous to the *PHO5* gene in *S. cerevisiae* (Orkwis *et al.* 2010; Kerwin and Wykoff 2012). *C. glabrata* does not contain any homologs of *ScPHO5* based on sequence similarity. *CgPMU1* appears to encode a narrow-range phosphatase, which is likely similar to the ancestral gene conserved in most Ascomycetes. *Cg*Pmu3 appears to have some functions in common with *Cg*Pmu2, but it is unknown what selective pressures led to the preservation of *CgPMU3*. *ScPHO5* also has a number of paralogs in *S. cerevisiae*, including *ScPHO3*, which has been suggested to be important for thiamine recycling through its activity as a thiamine phosphatase (Nosaka *et al.* 1989b, 1989a). This led us to explore thiamine metabolism in *C. glabrata*.

Thiamine and its metabolically active form, thiamine pyrophosphate (or TPP), are essential for a number of core intermediary carbon metabolism reactions (Gibson *et al.* 2016). TPP allows for decarboxylation of pyruvate, and was originally isolated as a cofactor for pyruvate dehydrogenase (Lipschitz *et al.* 1938). In *S. cerevisiae*, thiamine starvation activates the transcription factor *Sc*Thi3, which, together with its transcriptional co-activators, *Sc*Thi2 and *Sc*Pdc2, induces the expression of many genes related to the biosynthesis of thiamine. *C. glabrata* has subtle differences in how it synthesizes, recycles, or transports thiamine relative to *S. cerevisiae* (Iosue *et al.* 2016). *C. glabrata* lacks the Thi2 transcriptional coactivator in *S. cerevisiae*, lacks the ability to synthesize a pyrimidine precursor for thiamine synthesis, and in standard laboratory medium is a thiamine and pyridoxine (vitamin B_6_) auxotroph (Iosue *et al.* 2016). However, both species have many of the same biosynthetic genes, and those genes are regulated by the transcription factors, Pdc2 and Thi3. These subtle alterations are likely influenced by the environments of the two species (Gabaldón Estevan *et al.* 2013; Gabaldón *et al.* 2016).

We performed RNA-seq on *C. glabrata* cells to identify the genes that are regulated by thiamine starvation and identified *CgPMU3* as a highly induced gene during thiamine starvation. The identification of an analogous system to *S. cerevisiae* in *C. glabrata*, where there is a phosphatase that appears to be important for phosphate starvation responses and a paralog that is important for thiamine starvation responses, led us to explore whether there has been convergent evolution in multiple species to duplicate and specialize the phosphatase genes. Indeed, in *S. pombe*, there are two *ScPHO5* related phosphatases encoded by *SpPHO1* and *SpPHO4*, and transcriptional induction of these genes is dependent on different environmental conditions. *SpPHO1* is upregulated during phosphate and adenine starvation (Henry *et al.* 2011; Estill *et al.* 2015), and *SpPHO4* transcription is repressed by addition of thiamine to the medium (Yang and Schweingruber 1990; Yang *et al.* 1991). Given the similarities in behavior of paralogs, we characterized this behavior more fully to understand how flexible the evolutionary architecture for generating two phosphatases that are tailored to two different environmental conditions was.

## Materials And Methods

### RNA-seq

*C. glabrata* wild-type and *Cgthi3*Δ strains (Table S2) were grown in thiamine replete conditions at 30° to logarithmic growth phase (OD_600_ ∼0.2–0.5). Cells were harvested by centrifugation, washed three times with water, and transferred to SD medium (Sunrise Science, CA) with thiamine (thiamine replete: 0.4 mg/L) and without thiamine (thiamine starvation) and grown at 30°. *C. glabrata* wild-type was grown for 2 and 4 hr while the *Cgthi3*Δ strain was grown for 4 hr. This resulted in six samples: *C. glabrata wild-type* at 2 h, *C. glabrata wild-type* at 4 h, and *Cgthi3*Δ at 4 h, with thiamine replete and starvation conditions for all. RNA was harvested from these six samples for next generation sequencing. Eurofins Genomics generated an Illumina specific library for sequencing, performing 2x50 bp sequencing on a HiSeq2000, and yielding 92 million reads for the six samples. We utilized the Geospiza (Perkin Elmer) bioinformatics suite to perform analyses after reads per kilobase per million (rpkm) were grouped to NCBI annotated coding sequences of the *C. glabrata CBS138* genome. Two percent of genes change expression twofold in response to thiamine starvation (in either 2h or 4h). An .xls file is included (Table S1) with the expression (rpm) per gene both logarithm transformed and as rpkm counts. The raw FASTQ files are submitted to NCBI under accession SRP131893.

### Flow Cytometry

To assay induction of *C. glabrata PMU2* and *PMU3* using flow cytometry, we constructed plasmids where the full-length promoters of these genes (3 Kb *CgPMU2*p and 1 Kb *CgPMU3*p) were driving expression of YFP. The promoter sequences were amplified by PCR (Table S3) then repaired into a plasmid containing YFP by homologous recombination (Corrigan *et al.* 2013). These plasmids were transformed into *C. glabrata* wild-type, *Cgthi3*Δ (Iosue *et al.* 2016), and *Cgpho4*Δ (Kerwin and Wykoff 2009) strains (Table S2). Cells were grown at 30° in thiamine/phosphate replete medium to logarithmic growth phase (OD_600_ ∼0.2-0.5). Cells were harvested by centrifugation, washed 3 times with water, inoculated into thiamine replete (0.4 mg/L) and starvation and phosphate replete (1 g/L) and starvation conditions, and grown at 30° overnight (∼18 h). Fluorescence (in arbitrary units) of each strain was measured using a flow cytometer with a 533/30 FL1 filter set (Accuri C6, BD Biosciences).

### Phylogenetic Analysis

We downloaded *PHO5* homologs from 23 species (Wapinski *et al.* 2007). These species represent all major clades of the Ascomycota phylum, including the *Saccharomycotina* (budding yeast), *Pezizomycotina* (filamentous fungi), and *Schizosaccharomycetes* (fission yeast), although sampling is biased toward the first group, with the latter two being represented by just two and three species, respectively. To identify any homologs missing from the Fungal Orthogroup Repository, we performed BLAST searches on the following online databases: Saccharomyces Genome Database, Candida Genome Database, and Genome Resources for Yeast Chromosomes. In addition, we used HMMER webserver (https://www.ebi.ac.uk/Tools/hmmer/) to search against the Reference Proteomes to leverage the high sensitivity of the HMM-based tool for identifying distant homologs (Finn *et al.* 2015). For three species in the *sensu stricto* group (*S. paradoxus*, *S. mikatae*, *S. bayanus*), we downloaded the annotated FASTA file containing gene features from the Saccharomyces *sensu stricto* genome website (Scannell *et al.* 2011), and performed local BLAST searches. In total, we identified 53 putative *PHO5* homologs in the 23 species. The number of homologs per species ranges between zero (*C. glabrata*, *N. castellii*, and *E. gossypii*) to five (*K. lactis* and *S. cerevisiae*), with a median of 2 and a mean of 2.3 per species.

The amino acid sequences were aligned using ProbCons with the following parameters: 2 consistency reps, 1000 iterative refinement reps, 0 pre-training reps (Do *et al.* 2005). Protein phylogeny was reconstructed using PhyML website service (v3.0), with the aligned protein sequences as input and the following parameters: AIC for model selection, using BIONJ to construct the initial tree, SPR for tree improvement, no random starting tree used, 1000 bootstraps for Figure 3 and only 100 bootstraps for Figure S3, due to the much larger sample size (Guindon *et al.* 2010).

For Figure S3, we additionally performed gene-tree and species-tree reconciliation using an algorithm-based approach implemented in Notung (v2.9) (Durand *et al.* 2006). We first loaded the gene tree from the last step and a species tree based on a 1233-gene data matrix from (Shen *et al.* 2016). Reconciliation was performed with the default settings. The resulting gene tree was rooted by selecting the branch that minimized the total event score, following the software’s recommendation. Finally, the gene tree was “rearranged” using the Rearrangement function, which swaps weakly supported branches to identify the alternative topology that minimizes the total event score. We followed the software’s default setting to define weakly supported branches as those with less than 90/100 bootstrap values. This reduced the total number of duplications and losses from 29/48 to 23/15. Figure S3 shows the reconciled and rearranged gene tree with inferred duplication and loss events.

### qPCR Assay

Cells were grown and washed as described for flow cytometry but inoculated into thiamine/phosphate replete, thiamine starvation, and phosphate starvation conditions for 4 h at 30°. RNA was extracted with the Direct-zol RNA Miniprep Plus kit (Zymo Research) and reverse transcribed to cDNA using the iScript cDNA synthesis kit (Bio-Rad). cDNA was amplified in a 25 µL reaction using the Sso Advanced Universal SYBR Green Supermix (Bio-Rad) with a CFX quantitative PCR machine (Bio-Rad). Primers were designed to amplify phosphatase genes and *ACT1* in various species (Table S3). Transcript amounts for each gene were normalized to *ACT1*, the expression of which does not change in different thiamine and phosphate conditions (Kerwin and Wykoff 2009; Iosue *et al.* 2016). Ten-fold genomic DNA dilutions were also amplified with each primer set as amplification controls.

### Hydrolysis of TPP, PNPP, and 1-Napthyl-Phosphate

To assay hydrolysis of TPP in various yeasts, *C. glabrata PMU3* was deleted and precisely replaced with phosphatase genes using a *CgURA3* selection/counter selection scheme with 5-FOA. Multiple transformants were confirmed by PCR and tested in assays to verify that the observed phenotype was not an outlier. Strains (Table S2) were grown overnight in thiamine/TPP replete conditions. Cells were harvested and washed as described for flow cytometry and inoculated at a low density (OD_600_ = 0.001) into TPP replete (30 mg/L), thiamine replete, and thiamine/TPP starvation conditions and grown at 30° for 24 h. Cell density (OD_600_) was measured to indirectly assay the ability to hydrolyze TPP.

Because the *CgPMU3* promoter does not highly express genes under phosphate starvation, we cloned the phosphatase ORFs under the control of a phosphate-regulated promoter (*ScPHO5*) (Orkwis *et al.* 2010). The same genes from the TPP assay were recombined onto a *ScPHO5* promoted plasmid through gap repair and transformed into *Cgpmu2*Δ (Corrigan *et al.* 2013). *CgPMU2* was deleted to reduce background phosphatase activity. Strains (Table S2) were grown overnight in phosphate replete conditions, then cells were harvested and washed as for flow cytometry. Cells were inoculated into phosphate starvation conditions at a cell density of OD_600_ ∼0.1 and grown for ∼18 h. Cells were assayed for PNPP hydrolysis as previously described in (Kerwin and Wykoff 2009; Orkwis *et al.* 2010). Cells growing on agar plates lacking phosphate were assayed for hydrolysis of 1-napthyl phosphate as described in (Kerwin and Wykoff 2009; Orkwis *et al.* 2010).

### Supplementary Material and Data Availability

Supplementary data are provided in a single .xls file. Strains are available upon request. Raw FASTQ files are submitted to NCBI under accession SRP131893. Supplemental material available at Figshare: https://doi.org/10.25387/g3.6220109.

## Results

### RNA-seq of Thiamine Starved C. glabrata Identifies CgPMU3 as a Highly Induced Phosphatase

**Figure 1:**
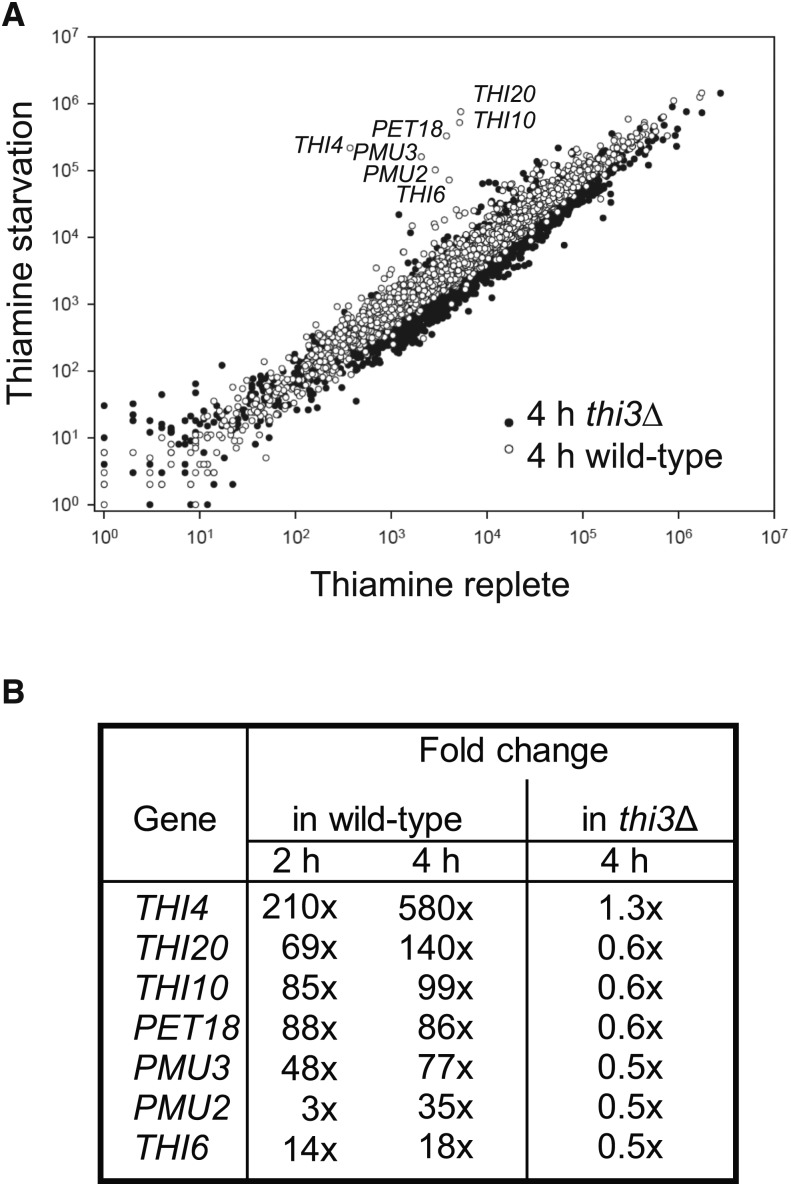
RNA-seq of thiamine-starved *C. glabrata*. (A) Normalized abundance of transcripts plotted for wild-type *C. glabrata* and a *Cgthi3*Δ strain that were grown for 4 h in thiamine starvation relative to cells grown in thiamine replete medium (strains grown in singlicate for this experiment). Transcripts that increased in abundance during starvation are visualized as circles that are above the majority of the genes. The seven most highly induced genes are *CgTHI3*-dependent. (B) A list of the most highly induced genes during thiamine starvation and their fold induction relative to the thiamine replete sample. Wild-type cells were grown for 2 h and 4 h to investigate the timing of induction; however, only the 4 h time point is presented in part (A).

To understand the transcriptional response of *C. glabrata* to thiamine starvation, we performed RNA-seq analysis on cells that were grown in medium either containing or lacking thiamine and identified genes up-regulated by thiamine starvation that were dependent on the transcriptional regulator *CgTHI3* (Figure 1). There is large overlap with the orthologous genes in *S. cerevisiae*, when examining a microarray of thiamine starvation in *S. cerevisiae* (Nosaka *et al.* 2005), including *THI4*, *PET18*, *THI20*, *THI6*, and *THI10* (Table S1), and we confirmed a statistically significant increase in these transcripts during thiamine starvation using qPCR on separately grown samples in triplicate (Figure S1). However, numerous genes induced in *S. cerevisiae* (*SNO*/*SNZ*/*THI5*/*THI2*/*PHO3*) lack orthologs in the *C. glabrata* genome. To determine any previously unidentified genes that might serve an analogous function in *C. glabrata* as in *S. cerevisiae*, we examined genes that were induced during thiamine starvation but did not have a known role in thiamine metabolism in *C. glabrata*. We noted that there was *CgTHI3*-dependent induction of *CgPMU2* and *CgPMU3* during thiamine starvation. We had previously identified *CgPMU2* as a *ScPHO5* analog, which is highly induced during phosphate starvation (Orkwis *et al.* 2010). Compared to *CgPMU2*, *CgPMU3* is induced more strongly and at an earlier time point (Figure 1). Given this observation, we hypothesize that *CgPMU3* functionally replaced the *PHO3* gene, which is missing from the *C. glabrata* genome.

### CgPMU2 and CgPMU3 Are Regulated By Two Different Environmental Conditions

**Figure 2:**
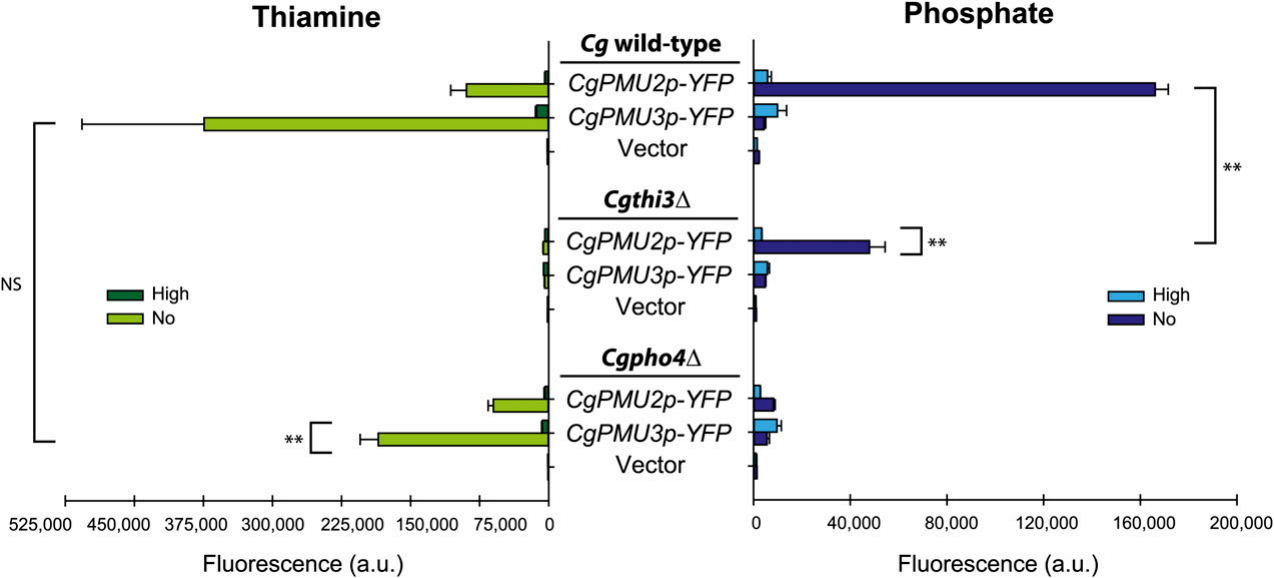
*CgPMU3* is tightly regulated by thiamine whereas *CgPMU2* is regulated by both thiamine and phosphate. Plasmids containing the full-length promoter of either *CgPMU2* or *CgPMU3* driving expression of *YFP* were transformed into *C. glabrata* wild-type, *thi3*Δ, and *pho4*Δ strains and assayed for fluorescence using flow cytometry. Error bars represent the standard deviation of three biological replicates. We performed a Student *t*-test to confirm that *CgPMU2* expression declined in the *Cgthi3*Δ strain; however, there is still a significant increase in expression relative to the high phosphate conditions. We additionally confirmed no statistical decline in the *Cgpho4*Δ strain during thiamine starvation in the *CgPMU3p-YFP*. The ** indicates a p value less than 0.001 and NS indicates a p value higher than 0.05. (Left) Strains were grown in thiamine replete (High) and starvation (No) conditions. Both *CgPMU2* and *CgPMU3* promoters are induced by thiamine starvation and this induction is regulated by *Cg*Thi3. (Right) Strains were grown in phosphate replete (High) and starvation (No) conditions. Only the *CgPMU2* promoter is induced by phosphate starvation and this induction is regulated by *Cg*Pho4.

To confirm the transcriptional regulation that we observed in the RNA-seq experiment with *CgPMU2* and *CgPMU3*, we constructed *CgPMU2*- and *CgPMU3*-promoted *YFP* plasmids and assessed their ability to recapitulate phosphate- (*PMU2*) and thiamine- (*PMU3*) repression of *YFP* expression in cells (Figure 2). We also tested expression of each promoter-*YFP* construct in the alternate repressing condition. *CgPMU3* is tightly regulated by thiamine conditions (phosphate starvation has little effect), whereas *CgPMU2* is de-repressed by both thiamine and phosphate starvation. Additionally, the induction of phosphatases in the two conditions requires the appropriate transcription factor –*i.e.* *Cg*Thi3 regulates thiamine starvation induction and *Cg*Pho4 regulates phosphate starvation induction. It is worth noting that we see some defect in the appropriate induction of a phosphatase when the other transcription factor is absent – *e.g.*, *CgPMU2-YFP* is not fully induced during phosphate starvation in the *Cgthi3*Δ strain, suggesting some potential cross-talk between the transcription factors. Additionally, because the *PMU1* gene family is in tandem in the *C. glabrata* genome, one might argue that this cross-talk is a consequence of relaxation of chromatin structure. However, we eliminated this possibility because the plasmids used did not contain any part of each other’s promoters, and these plasmids are likely not exposed to the same chromatin structure as in the genome. We conclude that *CgPMU3* transcription is tightly regulated by thiamine concentration, and *CgPMU2* is primarily induced during phosphate starvation, but partially upregulated in response to thiamine starvation.

### Repeated Expansion of Gene Families Encoding Phosphatases in the Saccharomycetaceae

Despite similar specialization of *CgPMU2* and *CgPMU3* *vs.* *ScPHO5* and *ScPHO3*, the *PHO5*-related genes are not evolutionarily related to the *PMU1*-related genes (Orlando *et al.* 2015). Therefore, the gene family expansion and the subsequent specialization among the genes occurred independently in these two species. This led us to hypothesize that such expansion and specialization of phosphatase-encoding genes has occurred repeatedly during the evolution of this group of diverse yeasts.

**Figure 3:**
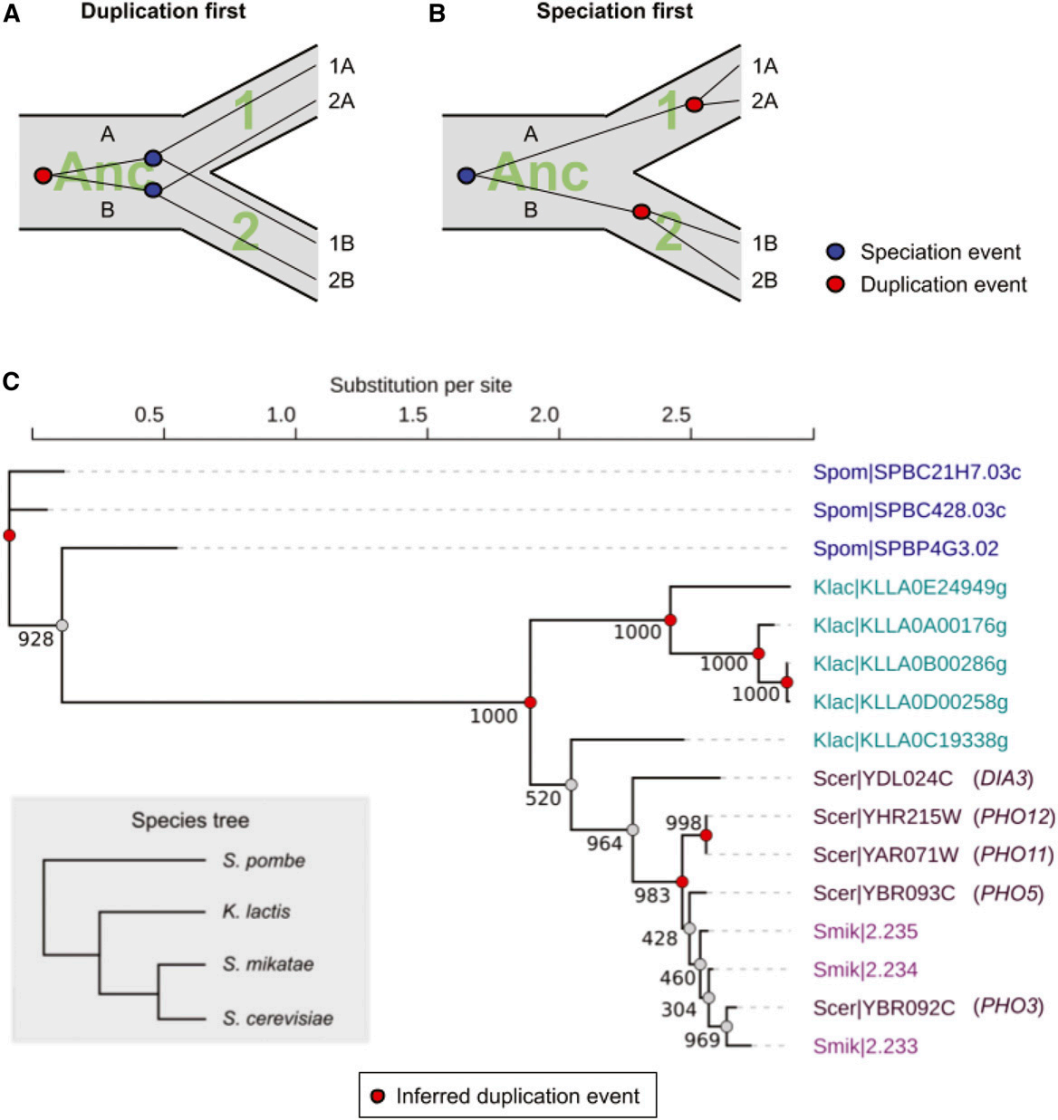
Repeated, relatively recent duplications of *PHO5* family in the Ascomycetes. **(**A) and (B) Cartoon example for the two evolutionary scenarios: (A) gene duplication precedes speciation, resulting in the orthologs (1A and 2A, 1B and 2B) to cluster first before they were joined by the duplication event; (B) speciation precedes gene duplication, resulting in the paralogs (1A and 1B, 2A and 2B) to cluster first before they were joined by the speciation event. Red and blue dots indicate gene duplication events and speciation events, respectively. (C) Maximum likelihood tree inferred based on protein sequence alignment of *PHO5* homologs in four species of Ascomycete yeasts. Numeric values next to the internal nodes indicate bootstrap values in support of the phylogeny shown (1000 replicates run). Red dots indicate inferred gene duplication events at nodes with strong bootstrap support (> 980/1000). The inset shows the species phylogeny. Gene loss events are not shown in this tree but are in Figure S3.

To test this hypothesis, we reconstructed the evolutionary history of the *PHO5* gene family in four *Ascomycota* yeasts that span a wide range of evolutionary distance, and which contain multiple members of the *PHO5* family. Three of the four species belong to the class of *Saccharomycetes*, including *S. cerevisiae*, *S. mikatae*, and *K. lactis*; the fourth species is *S. pombe*, which comes from the distantly related class of *Schizosaccharomycetes*, *i.e.*, the fission yeast. Under our hypothesis, there should be repeated, independent duplications that happened after speciation. To infer the relative timing of duplication to speciation, we reconstructed the gene tree for the *PHO5* homologs in these four species and compared it to the species tree. In a cartoon example with two paralogs in two extant species–designating the two paralogs as A and B, and the two species as 1 and 2–if gene duplication preceded speciation, gene 1A and 2A would cluster before they are joined by the cluster of 1B and 2B (Figure 3*A*). Conversely, if gene duplication occurred after speciation, 1A and 1B would coalesce first, and so would 2A and 2B, before the two clusters would coalesce (Figure 3*B*).

The maximum likelihood tree for *PHO5* family genes in these species strongly supports independent duplication in *S. pombe*, *K. lactis*, and the *sensu stricto* clade, which includes *S. cerevisiae* (Figure 3*C*). Within the closely related *sensu stricto* clade, which is estimated to have speciated in the past ∼20 million years, the protein sequence divergence between homologs was often too close for confident reconstruction of the phylogeny, as shown by the low bootstrap values in some of the internal nodes. Nonetheless, there is evidence for recent duplications. Examining the syntenic relationships, *e.g.*, for the genomic region that contains *ScPHO5* and *ScPHO3* arranged in tandem, we found that the syntenic region in *S. mikatae* contains three genes instead of two, one of which, S.mik|2.235, appears to have no orthologs in other species, suggesting a recent origin in that species (Figure S2). Similarly, *ScPHO11* and *ScPHO12* appear to have no syntenic orthologs in the other *sensu stricto* species.

We next asked if this pattern of repeated duplications post speciation is general across the Ascomycota fungi. To do this, we extended our analysis to 53 homologs of *PHO5* in 23 Ascomycota yeasts (Wapinski *et al.* 2007; Gabaldón *et al.* 2016; Shen *et al.* 2016). For this large dataset, we used an algorithm-based approach to reconcile the gene tree with the species tree, allowing for duplications and losses. The result revealed extensive duplication and loss events not just at the tip of the tree, but rather throughout the species phylogeny (Figure S3). There were both ancient and recent duplications, accompanied by multiple loss events, revealing an incredibly dynamic history of the phosphatase gene family. In conclusion, phylogenetic analysis confirmed our hypothesis that the *PHO5* family has experienced repeated duplication (and loss) in multiple yeast species in the *Ascomycota* phylum.

### Multiple Yeast Species Specialize the Transcription of Phosphatase Genes

Our data in combination with the phylogenetic analysis raised the question of whether multiple yeast species have this same specialization behavior –*i.e.* a phosphatase repressed by thiamine and a phosphatase repressed by phosphate. Others have observed similar partitioning of phosphatases in *S. cerevisiae* and *S. pombe*, but none have directly compared the two nutrient conditions side-by-side (Yang and Schweingruber 1990; Henry *et al.* 2011).

**Figure 4:**
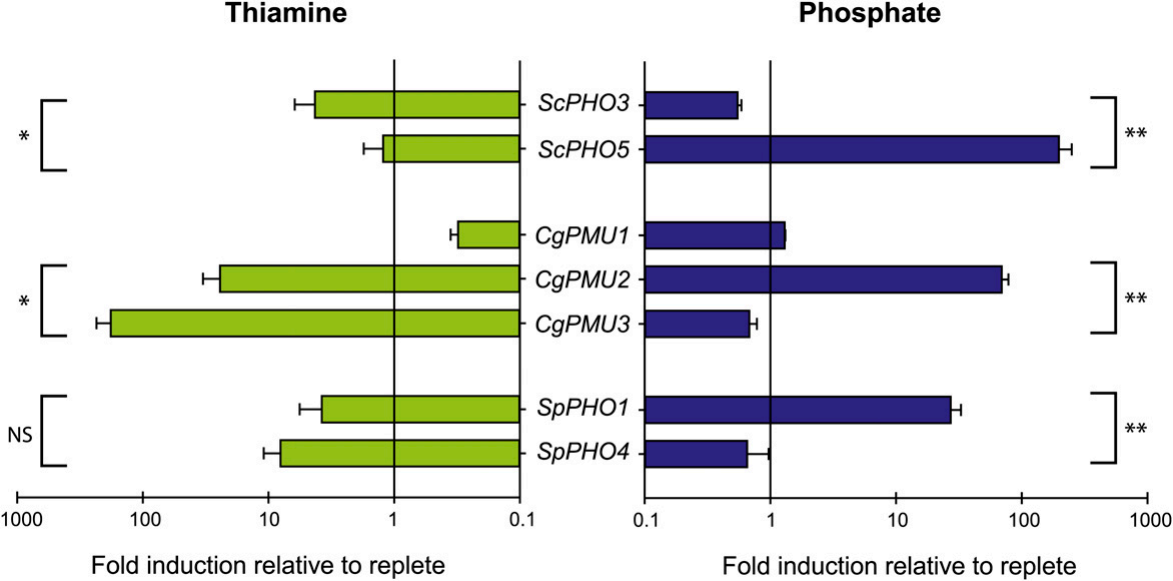
Phosphate-repressible phosphatases are more tightly regulated than thiamine-repressible phosphatases. *S. cerevisiae*, *C. glabrata*, and *S. pombe* wild-type strains were grown in replete, thiamine starvation, and phosphate starvation conditions. qPCR on reverse-transcribed RNA from these strains determined the amount of transcript for various phosphatase genes. Transcript levels were normalized to transcript levels for *ACT1*, which does not change its expression in response to nutrient conditions, and fold induction was calculated relative to replete for both thiamine (Left) and phosphate (Right) starvation conditions. Error bars represent the standard deviation of the average of fold induction for three independently grown biological replicates. We compared with Student *t*-tests the two paralogs in each condition, and the p value below 0.05 is indicated with one * and 0.001 with **. If the p value was above 0.05 it was considered not significant (NS). (Left) Thiamine-repressible phosphatases are not tightly regulated, as the other paralogs show some induction. (Right) Phosphate repressible phosphatases are tightly regulated, as there is no induction of the other paralogs.

To understand the transcriptional regulation of duplicate phosphatase-encoding genes in response to thiamine and phosphate starvation, we used qPCR of reverse-transcribed RNA from cells grown in nutrient replete, thiamine-starved, and phosphate-starved conditions, and assessed the abundance of phosphatase transcripts in *S. cerevisiae*, *C. glabrata*, and *S. pombe* (Figure 4). We find there is tight regulation of the phosphate-repressible phosphatases. Only *ScPHO5*, *CgPMU2*, and *SpPHO1* are highly induced by phosphate starvation. The thiamine-regulated paralogs show no induction during phosphate starvation (Figure 4**, Right**). Gene induction during thiamine starvation is not as tightly regulated. The thiamine-repressible phosphatases, *ScPHO3*, *CgPMU3* and *SpPHO4* are induced (note the log scale in the *x*-axis) during thiamine starvation. However, *CgPMU2* and *SpPHO1* show some induction as well (Figure 4**, Left**), and only *S. cerevisiae* and *C. glabrata* have statistically significant differential paralog regulation, although in *S. pombe* the trend is present (but not statistically significant at a p value of 0.09). In general, we observe some repression of one paralog by thiamine, and another paralog strongly by phosphate, supporting our hypothesis that multiple yeast species have partitioned the transcription of one phosphatase for each condition.

### Substrate Specialization in Both the *PHO5* and *PMU1* Phosphatase Protein Families

**Figure 5:**
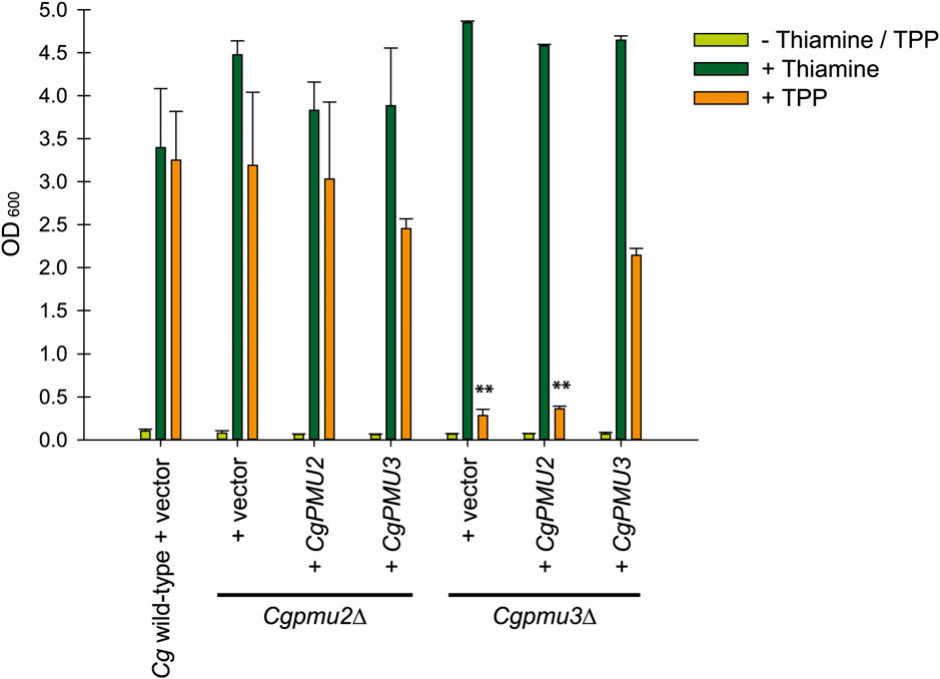
*CgPMU3* encodes a functional thiamine pyrophosphatase (TPPase). *C. glabrata* wild-type, *pmu2*Δ, and *pmu3*Δ strains, containing an empty vector, were grown in thiamine/TPP starvation, thiamine replete, and TPP replete conditions. Plasmids containing *CgPMU2* or *CgPMU3* under the control of the *ScPHO5* promoter were introduced into the deletion strains and grown in the same conditions. The *ScPHO5* promoter expresses at a low level regardless of thiamine concentration in *C. glabrata* (Orkwis *et al.* 2010). Optical density at 600 nm was measured to determine growth. All strains grow to a high density when thiamine is supplied. *Cgpmu3*Δ shows a defect when TPP is the sole source of thiamine and only addition of *CgPMU3* rescues this defect. Error bars represent the standard deviation of the average of three independently grown biological replicates. The ** indicate a p value below 0.001 when those samples are compared to wild-type with TPP, and all of the other TPP samples are not statistically significantly different from one another.

Since we observed specialization of the phosphatases in terms of initiation of transcription, we explored whether the phosphatases had specialized their activity toward substrates –*i.e.* are the phosphatases that are induced during thiamine starvation well-tailored to TPP as a substrate? When cells die, they often release the active cofactor TPP, and in order for the thiamine to be accessed it must be hydrolyzed to thiamine, as thiamine transporters appear to be unable to transport TPP (Nosaka *et al.* 1989a). We expected that if *CgPMU3* encodes a phosphatase required for the hydrolysis of TPP to thiamine, a *Cgpmu3*Δ strain might not utilize TPP as a thiamine substrate. Because *C. glabrata* behaves as a thiamine auxotroph in standard growth medium, we expected that a lack of TPP hydrolysis to release thiamine could limit growth, as measured by optical density. Indeed, deletion of *CgPMU3* led to a defect in growth when TPP was supplied as the sole thiamine source, whereas deletion of *CgPMU2* did not (Figure 5). We introduced plasmids containing the phosphatases under the control of a low-level, non-thiamine regulated promoter (Orkwis *et al.* 2010). Expressing *CgPMU3* in a *Cgpmu3*Δ suppressed this defect, but *CgPMU2* did not (Figure 5).

**Figure 6:**
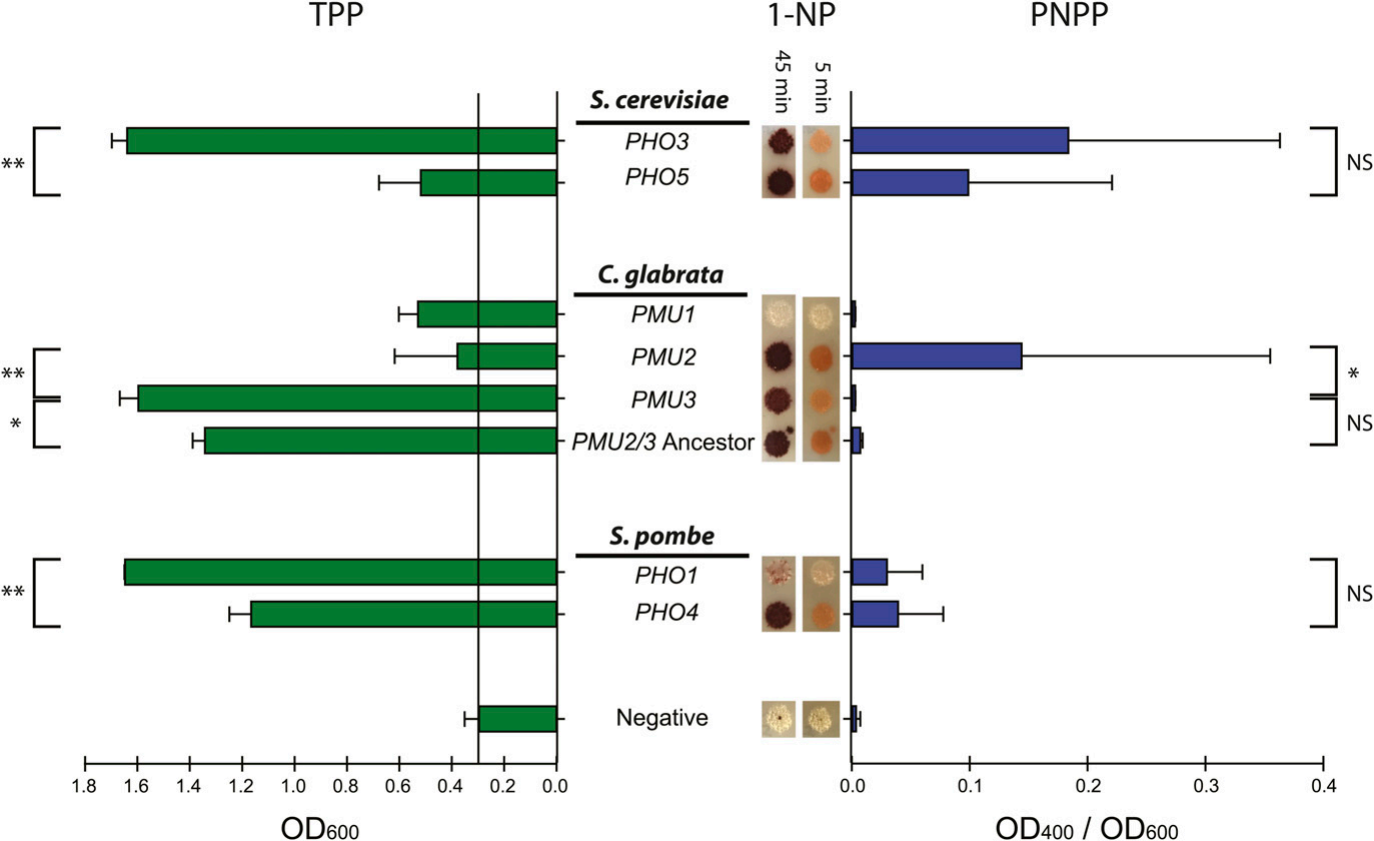
*PHO5* and *PMU1* protein family members have different specificities against substrates - some are better TPPases and others are more broad-range organic phosphatases. (Left) The *CgPMU3* ORF was deleted and replaced with ORFs of phosphatases from various yeasts so that they are under the control of the *CgPMU3* promoter and inducible in growth medium containing TPP as the sole thiamine source. The ability to hydrolyze TPP to thiamine was assayed by measuring growth using optical density at 600 nm. (Right) The phosphatase ORFs were cloned into plasmids under the control of the *ScPHO5* promoter, which is highly expressed during phosphate starvation, and these plasmids were introduced into a *Cgpmu2*Δ, which has minimal PNPPase activity. Cells were assayed for PNPP hydrolysis, indicated by an increase in OD_400_/OD_600_. (Right, images) The strains containing the plasmids were grown on solid medium lacking phosphate and assayed for 1-napthyl-phosphate hydrolysis, which is indicated by a red color. Activity was assayed after 5 min and 45 min. For the bar graphs, error bars represent the standard deviation of the average of three to six replicates. We compared with Student *t*-tests the two paralogs in each condition, and a p value below 0.05 is indicated with one * and 0.001 with **. If the p value was above 0.05 it was considered not significant (NS).

The previous experiment did not eliminate the possibility that *CgPMU2*, when highly expressed, was capable of hydrolyzing TPP at a low rate which could allow for growth. To test for this possibility, as well as to test the other known phosphatases for the ability to cleave TPP at a physiological level, we precisely deleted the *CgPMU3* open reading frame (ORF) with the *CgURA3* gene and then subsequently replaced the *CgURA3* gene with an ORF of our choosing, through 5-FOA selection. This allowed for the ORF to be regulated by the endogenous *CgPMU3* promoter and determined whether high level induction of *CgPMU2* would allow for physiological hydrolysis of TPP (Figure 6**, Left**). *CgPMU2* does not encode a phosphatase that recognizes TPP at physiological conditions; thus, *Cg*Pmu2 and *Cg*Pmu3 have different functional specificities *in vivo*. As expected, in the *PHO5* phosphatase gene family, we observe high activity for TPP with the phosphatase (*ScPHO3*) that is induced during thiamine starvation.

We were also curious whether the phosphatases might have altered specificity toward other substrates. To test this hypothesis, we measured the activity of the phosphatases toward commonly used laboratory organic phosphates such as p-nitrophenyl phosphate (PNPP) and 1-napthyl phosphate (1-NP) (Orlando *et al.* 2015) in addition to TPP hydrolysis. We first cloned each phosphatase ORF under the control of the *ScPHO5* promoter in a plasmid so that the phosphatase would be highly expressed during phosphate starvation (Orkwis *et al.* 2010). These plasmids were then transformed into a *Cgpmu2*Δ strain to remove native PNPPase activity, as this strain has very low phosphate-starvation phosphatase activity (Orkwis *et al.* 2010). Because we used the same plasmid, promoter, and growth conditions, we expected that roughly the same amount of protein would be present regardless of ORF. We then measured the ability to cleave phosphate from PNPP in whole cells expressing the various phosphatases (Figure 6**, Right**). We observed differences between the paralogs of both the *PHO5* and *PMU1* families. The *PMU1* family of phosphatases appears to have tuned phosphatase activity such that the phosphate starvation-induced phosphatase (*Cg*Pmu2) is extremely efficient at hydrolyzing PNPP, and the thiamine starvation-induced phosphatase (*Cg*Pmu3) is extremely efficient at hydrolyzing TPP. However, in *S. cerevisiae*, both members of the *PHO5* family had significant PNPPase activity, while *Sc*Pho3 appears to be a more efficient TPPase.

We also assessed the ability of the cloned phosphatase genes to hydrolyze 1-NP. We have already observed differences in the *PMU1* family with regards to these two phosphatase substrates – both *Cg*Pmu2 and *Cg*Pmu3 are efficient at hydrolysis of 1-NP, but only *Cg*Pmu2 can hydrolyze PNPP (Orlando *et al.* 2015). We do not observe these same differences between paralogs in *S. cerevisiae* and *S. pombe*. PNPP and 1-NP have different physical structures, and our results hint that identifying the *in vivo* biological substrate of a given phosphatase may be difficult, as there may be multiple relevant substrates or the promiscuity of the enzyme for many substrates may prevent strong conclusions.

In *S. cerevisiae* and *S. pombe* there does not appear to be a loss of activity against PNPP or 1-NP in the thiamine starvation induced phosphatases (*Sc*Pho3 and *Sp*Pho4), as we observe in the *PMU1* family (Figure 6**, Right**). This might be explained by the fact that these two protein families have different amino acids present in the active site (Orlando *et al.* 2015), and the *PHO5* family is unable to discriminate between the organic compounds attached to the phosphate. Regardless, in *S. cerevisiae* the activity of the phosphatases against TPP are tailored for the phosphatase that is induced in thiamine starvation (*Sc*Pho3) (Figure 6**, Left**). Interestingly, we observe a different case in *S. pombe*. Both phosphatases appear to be capable of cleaving TPP, and the phosphate starvation-regulated phosphatase, *SpPHO1*, appears to be slightly better at cleaving TPP. In fact, the thiamine starvation-regulated *SpPHO4* appears to be a better phosphatase against 1-NP and PNPP relative to *SpPHO1*, which is counter to published data that indicates that *SpPHO1* is the phosphate starvation inducible phosphatase (Henry *et al.* 2011; Estill *et al.* 2015). However, the phenotype of lack of phosphatase activity in the *Sppho1*Δ strain is only observable in a medium mixture that contains thiamine, likely repressing *SpPHO4* expression (standard *S. pombe* medium does not contain thiamine, and the phosphatase assays were performed in a SD/EMM medium mixture) (Henry *et al.* 2011; Carter-O’Connell *et al.* 2012). Additionally, the phosphatase genes in *S. pombe* appear to be expressed at higher levels under repressing conditions (Henry *et al.* 2011), so *S. pombe* may have a different approach from the other two species studied. The contrast in approaches taken by the different species to deal with phosphate and thiamine starvation highlights two important points. First, some species’ phosphatases have a high degree of specialization at the level of the enzyme, while others do not, and second, because we are only looking at a few laboratory substrates, our results give guidance, but not strong conclusions, with regards to the *in vivo* functions of individual phosphatases.

### The Ancestor of CgPMU2 and CgPMU3 Is Most Similar to CgPMU3

Because *CgPMU2* and *CgPMU3* are more closely related to one another than to *CgPMU1*, we wanted to determine the enzyme specificity of the likely ancestor of *CgPMU2* and *CgPMU3*. To test the functional specificity of the ancestor, we generated a *Cg*Pmu1 protein where every amino acid that was common to *Cg*Pmu2 and *Cg*Pmu3 was introduced into the *Cg*Pmu1 background (Figure S4). We posited that common sequences in both *Cg*Pmu2 and *Cg*Pmu3 were likely to have been common in the ancestor of both. The ancestor appears to resemble *Cg*Pmu3 specificity for TPP (Figure 6). This result suggests that the primary selective condition for maintenance of the expansion of the *PMU1* gene family may have been to recycle thiamine as opposed to scavenge phosphate from organic phosphates; however, because we observe significant 1-NP hydrolase activity for the reconstructed ancestor protein, we cannot exclude recycling of phosphate as a selective force. These data are consistent with the *Cg*Pmu2 protein having neofunctionalized relative to its ancestor, and now being able to readily hydrolyze PNPP, losing the ability to hydrolyze TPP.

## Discussion

This work demonstrates that two different phosphatase gene families (the *PHO5* and *PMU1* families) experienced independent duplication events in multiple lineages, and that in both cases, the duplicates specialized at the transcriptional level to be responsive to either thiamine starvation or inorganic phosphate starvation. Additionally, this duplication in some species allowed for phosphatase specificity to be tailored for their respective substrates. An interesting evolutionary question is, under similar environmental pressures (or opportunities), does evolution repeatedly use the same raw genetic materials for adaptation? How predictable are evolution and adaptation? In our study, we found that *C. glabrata* lost the ancestral *PHO5* family and recreated the same functional specialization phenotype by co-opting an expanded *PMU1* family. This demonstrates that while the evolution of the phenotype is convergent (duplication of phosphatase genes and specialization), the genetic basis for the phenotype can be different (Stern 2013). In other cases, however, evolved phenotypes can appear to be highly constrained, and thus, predictable (Zhen *et al.* 2012). In extreme cases, limited evolutionary “solution space” can lead to parallel evolution involving the same amino acid changes in orthologs (Zhen *et al.* 2012). Our observation of multiple *Ascomycota* yeasts (*e.g.*, *S. cerevisiae*, *K. lactis*, and *S. pombe*) independently duplicating and specializing the *PHO5* family suggests that this family may be the preferred substrate for evolution, but when the *PHO5* family is not available (for example, in *C. glabrata*), other options can be explored. Such dynamism suggests multiple “solutions” to the problem.

Another question is, what might be driving the gene family evolutionary dynamics? Our observation of two different phosphatase families being involved in generating a convergent phenotype suggests the possibility that there is a common selective pressure favoring this specialization, although the multiple losses seem to suggest that the selection for maintaining this specialization is weak or variable across evolutionary timescales (Stern 2013). We cannot exclude the possibility that neutral processes generate this seemingly complex genomic architecture repeatedly (Force *et al.* 1999), and potentially in some cases, genetic drift may have mimicked this apparent convergence. However, with the *PMU1* family, the data are consistent with neofunctionalization, as the activities of *Cg*Pmu2 and *Cg*Pmu3 have diverged. The genetic architecture (especially with regards to transcriptional regulation) is reminiscent of the adaptive *GAL* switch in *S. cerevisiae* (Hittinger and Carroll 2007).

The convergent behavior has noticeable noise -*i.e.* not every species has tight transcriptional regulation, and the *PHO5* phosphatase family appears to have significant TPPase activity even in the phosphate starvation-regulated phosphatases. Because we observe this convergent behavior in *sensu stricto* species and the distantly related Archaeascomycete *S. pombe*, it seems possible that the unicellular fungal lifestyle common to these species has imparted a selective advantage to the partitioning of phosphatase expression and activity to a single environmental condition. Because thiamine is critical for energy metabolism, it is appealing to consider that phosphatase duplication and evolution may have been shaped by a need to conserve energy by engaging the most efficient response to recycle thiamine and/or to acquire phosphate from organic phosphate sources. It is worth noting that there are no obvious, clear links between phosphate and thiamine metabolism, as the signaling pathways are different, and there is no clear single point where the two pathways would intersect. There are likely indirect interactions between the two metabolic pathways in that ATP/energy charge may impact the phosphorylation of thiamine, and that energy metabolism is influenced by phosphate concentrations. If duplication and loss happen at a relatively high rate over evolutionary timescales, as Figure S3 suggests, one possibility is that having multiple, specialized copies may only provide a small advantage, and thus, the selective pressure to make the specialization complete at both the transcriptional and enzymatic level may be low.

The two phosphatase families (that share no sequence homology based on BLASTp alignments) appear to have different ancestral functions. Little is known about *PMU1*, other than most Ascomycetes have one copy of the gene, the gene is involved in the dephosphorylation of trehalose, and it contains a phosphomutase-like domain (Elliott *et al.* 1996; Orkwis *et al.* 2010). In *C. glabrata*, there were two duplication events, and after the first duplication event which generated the ancestor of *CgPMU2* and *CgPMU3*, the phosphatase acquired the ability to cleave TPP. After the second duplication event, *CgPMU2* specialized into a broad-range acid phosphatase, and *CgPMU3* maintained the TPPase activity. It is tempting to speculate that loss of the *PHO5* family and gain of the *PMU* family is related to the pathogenicity of *C. glabrata*, but the same expansion/contraction did not occur in the pathogen *C. albicans*, so there is little evidence to support this coincidence. We believe that the *PHO5* family ancestrally had both TPPase and some broad-range phosphatase activity. The dynamic duplication and gene loss events that occurred over evolutionary time allowed for likely rare cases of specialization – what we observe in *S. cerevisiae*. In *S. cerevisiae*, changes in the amino acid sequence of the phosphatase genes allowed for specialization toward TPP, and specialization toward other organic phosphatases. However, this specialization is only one possibility, as *S. pombe PHO5* homologs suggest that you can have both activities present at the same time (TPPase and PNPPase activity). It is worth emphasizing that we do not have access to the true ancestors, and so this evolutionary history is speculative in nature.

Often, phosphatases are thought of as releasing phosphate from an organic phosphate substrate, but we demonstrate that phosphatase expression might be more important for the recycling of the organic substrate (thiamine). In support of this argument are the results with a gene that mimics the ancestor of the *CgPMU2* and *CgPMU3* genes. This protein, which contains all of the amino acid changes common to *Cg*Pmu2 and *Cg*Pmu3 in the context of *Cg*Pmu1, has phosphatase substrate specificity that mirrors the thiamine-regulated *Cg*Pmu3 protein. Selection regimes that place energetic constraints on phosphatase expression and the recycling of thiamine, which is critical for sugar utilization, may have generated the behaviors that we observe in multiple Ascomycetes. Given the dynamic nature of the *PHO5* and *PMU1* gene families, it is not possible to ascertain what selective pressures drove the behaviors we observe, but this work highlights that recycling the organic compound connected to phosphate is also important.
